# Bilateral macronodular adrenocortical disease with isolated primary aldosteronism and normocortisolemia: a case report

**DOI:** 10.3389/fmed.2026.1806817

**Published:** 2026-07-15

**Authors:** Yaoqiang Ren, Zehong Ao, Min Wei, Xiaoli Yan, Xiaodong Wang

**Affiliations:** Department of Urology Surgery, Fenyang Hospital of Shanxi Province, Lüliang, Shanxi, China

**Keywords:** adrenalectomy, ARMC5 mutation, bilateral macronodular adrenocortical disease, primary aldosteronism, somatic mutation

## Abstract

Primary aldosteronism (PA) is a common cause of secondary hypertension. Bilateral macronodular adrenocortical disease (BMAD) with ARMC5 mutations is classically associated with hypercortisolism. This report describes a 54-year-old female with hypertension and hypokalemia, biochemically confirmed as PA, and imaging showing bilateral adrenal macronodules. Postoperative peripheral blood genetic testing identified a germline ARMC5 frameshift insertion (c.337dup). Whole-exome sequencing of the resected adrenal tissue revealed an additional somatic frameshift deletion (c.277_316del, p. P93fs, VAF 43.1%) absent in blood, demonstrating biallelic ARMC5 inactivation. Following unilateral adrenalectomy, the patient’s aldosterone levels normalized. This case shows that ARMC5 mutations can cause isolated mineralocorticoid excess without hypercortisolism, expanding the known phenotypic spectrum. Genetic testing for ARMC5 should be considered in patients with bilateral adrenal enlargement regardless of steroid hormone profile.

## 1 Introduction

Primary aldosteronism (PA) is a common cause of secondary hypertension and is associated with a higher cardiovascular and renal risk compared to essential hypertension (1). The diagnostic workup for PA, following a positive screening aldosterone-to-renin ratio, involves confirmatory testing and localization studies, with adrenal computed tomography (CT) serving as the initial imaging modality (2). The 2025 Endocrine Society clinical practice guideline recommends adrenal lateralization with CT and adrenal vein sampling (AVS) prior to surgical decision-making (3). A 2025 systematic review and meta-analysis reported that combining anatomical imaging with biochemical parameters (e.g., aldosterone >20 ng/dL, hypokalemia ≤3.5 mmol/L, renin ≤5 mIU/L) can achieve specificity up to 98% for identifying unilateral aldosterone hypersecretion, supporting the validity of imaging as the sole criterion for lateralization when combined with these biochemical features (4) Bilateral macronodular adrenocortical disease (BMAD), historically termed primary bilateral macronodular adrenal hyperplasia (PBMAH), is an entity typically manifesting as ACTH-independent Cushing’s syndrome due to autonomous cortisol secretion (5). Approximately 20–25% of sporadic cases and up to 80% of familial cases are driven by pathogenic germline mutations in the ARMC5 gene (6). ARMC5 is expressed in adrenocortical cells, bone marrow hematopoietic cells, and neurons and glial cells in the brain. The classic phenotype associated with ARMC5 mutations is one of hypercortisolism, ranging from overt Cushing’s syndrome to subclinical autonomous cortisol secretion, with bilateral adrenal macronodules being a common finding (7).

Genetically confirmed PBMAH2 with primary aldosteronism and no hypercortisolism is less common. This case of ARMC5-mutated BMAD presents with isolated mineralocorticoid excess without hypercortisolism, illustrating a phenotype not separately categorized in the 2022 WHO classification of adrenal cortical nodular diseases (8). In this atypical presentation, clinical suspicion for a genetic syndrome like PBMAH2 is lower in patients whose biochemical profile aligns with common sporadic primary aldosteronism rather than glucocorticoid excess. Consequently, such patients may undergo standard PA workup without proceeding to genetic evaluation, potentially missing a heritable condition with implications for family screening and long-term management.

## 2 Case description

### 2.1 Patient information

A 54-year-old female patient with a history of diabetes mellitus, managed with metformin extended-release tablets, was admitted to the Department of Cardiovascular Medicine at Shanxi Fenyang Hospital on December 4, 2025, due to poorly controlled hypertension over the preceding month. She had been diagnosed with hypertension in September 2021 and was on treatment with telmisartan and amlodipine besylate. Her blood pressure had been well-controlled until 1 month prior to admission, when hypokalemia also developed. Upon admission, her blood pressure was 199/110 mmHg.

### 2.2 Clinical findings

Initial laboratory investigations revealed hypokalemia with a serum potassium level of 3.08 mmol/L. Hormonal assessments showed an elevated aldosterone level. Specifically, the aldosterone-to-renin ratio (ARR) was increased in both supine and upright positions: 54.6 (aldosterone 259.362 pg./mL, renin 4.748 pg./mL) supine and 31.9 (aldosterone 333.555 pg./mL, renin 10.456 pg./mL) upright. Adrenocorticotropic hormone (ACTH) and cortisol levels at 8:00 a.m. were 8.824 pg./mL and 15.924 μg/dL, respectively, and at 4:00 p.m. were 7.024 pg./mL and 7.562 μg/dL, respectively. An adrenal ultrasound indicated space-occupying lesions in both adrenal glands. The patient was transferred to the Department of Urology on December 5, 2025, for further management.

### 2.3 Diagnostic assessment

**Figure 1:**
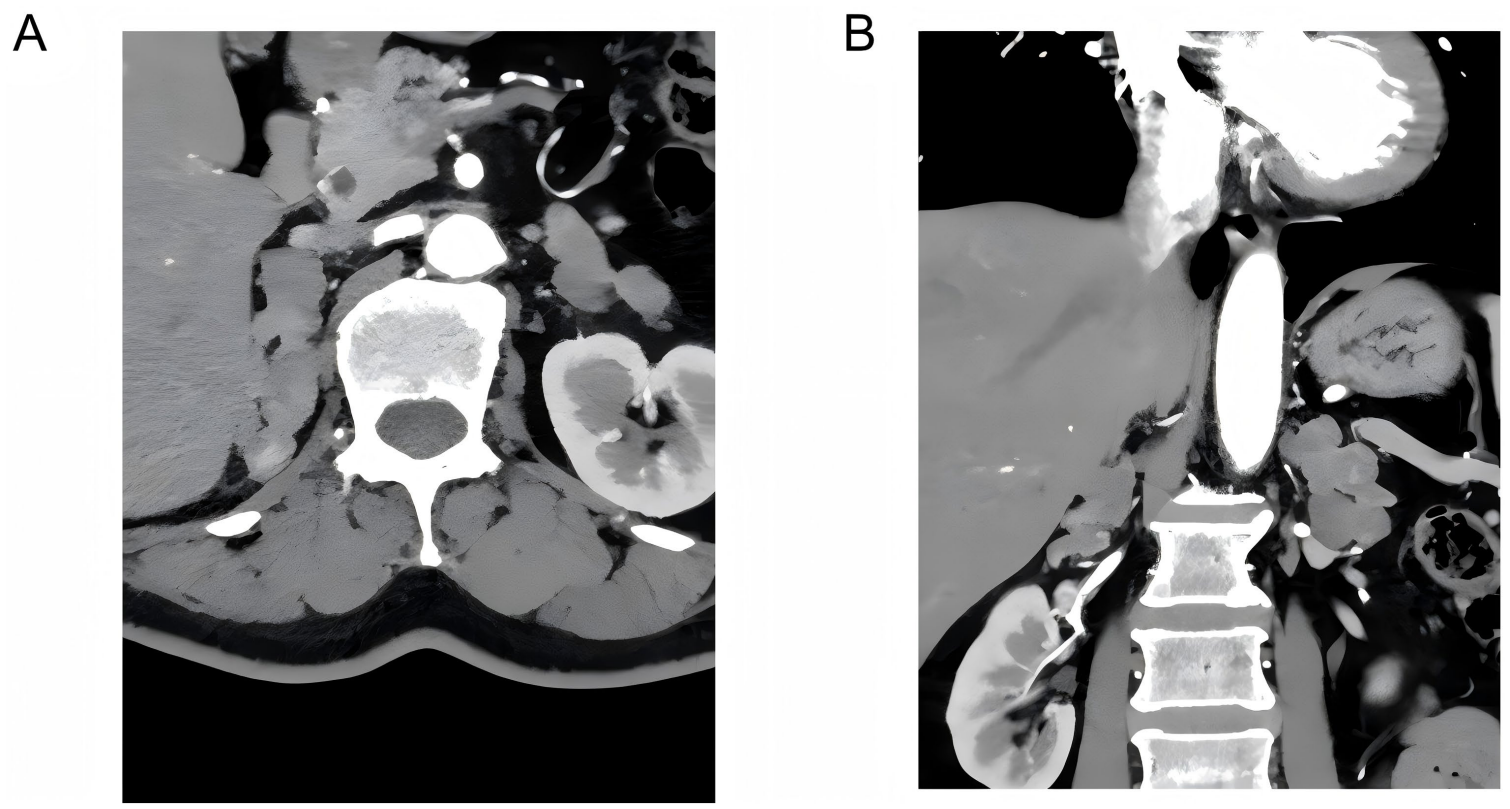
Preoperative computed tomography (CT) images of the abdomen demonstrating bilateral adrenal nodules. **(A)** Axial view. **(B)** Coronal view.

To exclude pheochromocytoma, plasma and 24-h urinary catecholamine profiles were measured via high-performance liquid chromatography–tandem mass spectrometry (HPLC-MS/MS). The results, including normetanephrine and 3-methoxytyramine levels, were within normal ranges, ruling out a catecholamine-secreting tumor. A low-dose (1 mg overnight) dexamethasone suppression test performed on December 12 yielded a positive result, with an 8:00 a.m. cortisol level of 8.505 μg/dL. A subsequent high-dose (8 mg overnight) dexamethasone suppression test on December 15 was negative, as the cortisol level measured 14.570 μg/dL the following morning, representing a suppression of less than 50%. Contrast-enhanced computed tomography (CT) of the abdomen confirmed bilateral macronodular adrenal hyperplasia (Figure 1). Magnetic resonance imaging (MRI) of the head revealed no evidence of pituitary or hypothalamic pathology. Based on the combination of hypertension, hypokalemia, an elevated ARR, and imaging findings of bilateral adrenal nodules, a diagnosis of primary aldosteronism due to bilateral macronodular adrenal hyperplasia was established.

### 2.4 Therapeutic intervention

Preoperative preparation aimed at controlling blood pressure and correcting hypokalemia was initiated. The regimen included spironolactone 60 mg every 8 h, telmisartan 40 mg daily, and levamlodipine besylate 2.5 mg daily, supplemented with intravenous and oral potassium replacement. Telmisartan was continued despite renin suppression because of its additional benefits in patients with diabetes and hypertension, including improved insulin sensitivity and renal protection. After 2 weeks of this therapy, blood pressure stabilized within the range of 130–140/80–90 mmHg, and serum potassium normalized to 4.6 mmol/L. Consultations with cardiology, neurology, endocrinology, and anesthesiology were completed to assess the patient’s fitness for surgery and formulate a contingency plan for intraoperative blood pressure fluctuations. The patient declined invasive AVS. She was informed that bilateral aldosterone secretion might persist after surgery, and she consented to the unilateral approach. Preoperative contrast-enhanced CT showed bilateral adrenal macronodules, with the largest nodule measuring 19 mm in diameter located in the left adrenal gland (Figure 1). The patient’s biochemical profile—aldosterone 259.362 pg./mL (equivalent to 25.9 ng/dL, >20 ng/dL) and hypokalemia (3.08 mmol/L, ≤3.5 mmol/L)—combined with CT imaging and laboratory findings, yielded a KASAI score >9, which has been reported to predict unilateral aldosterone hypersecretion with high specificity. Given that the left side was dominant among the bilateral adrenal nodules, the patient underwent a laparoscopic left adrenalectomy on December 20, 2025.

### 2.5 Follow-up and outcomes

Postoperative recovery was uneventful. Laboratory tests 1 week after surgery showed normalization of aldosterone levels and the ARR (Table 1). The postoperative ARR was 9.0 (aldosterone 71.319 pg./mL, renin 7.893 pg./mL) supine and 3.8 (aldosterone 82.943 pg./mL, renin 22.089 pg./mL) upright. Serum potassium remained normal at 4.51 mmol/L.

**Table 1:** Perioperative biochemical profile of the patient.

Characteristics	2025.12.05	2025.12.08	2025.12.10	2025.12.12	2025.12.17	2025.12.18	2025.12.21 (Postoperative day 1)	2025.12.27 (Postoperative week 1)	2026.02.03
Aldosterone (upright) (pg/ml**)**	333.555							82.943	103.052
Aldosterone (supine) (pg/ml)	259.362							71.319	83.920
Serum Potassium (mmol/l**)**	3.43	3.08	3.45	4.17	4.64	4.72	4.52	4.51	4.21
ACTH (8 h) (pg/ml)	8.824							9.816	10.468
Cortisol (8 h) (ug/dl)	15.924							9.256	16.376
ACTH (16 h) (pg/ml)	7.024							2.674	7.266
Cortisol (16 h) (ug/dl)	7.562							5.752	11.819
ACTH (24 h) (pg/ml)								1.775	1.465
Cortisol (24 h) (ug/dl)								5.727	4.455
Angiotensin II (upright) (pg/ml)	110.409							101.535	
Angiotensin II (supine) (pg/ml)	105.361							105.462	

**Figure 2:**
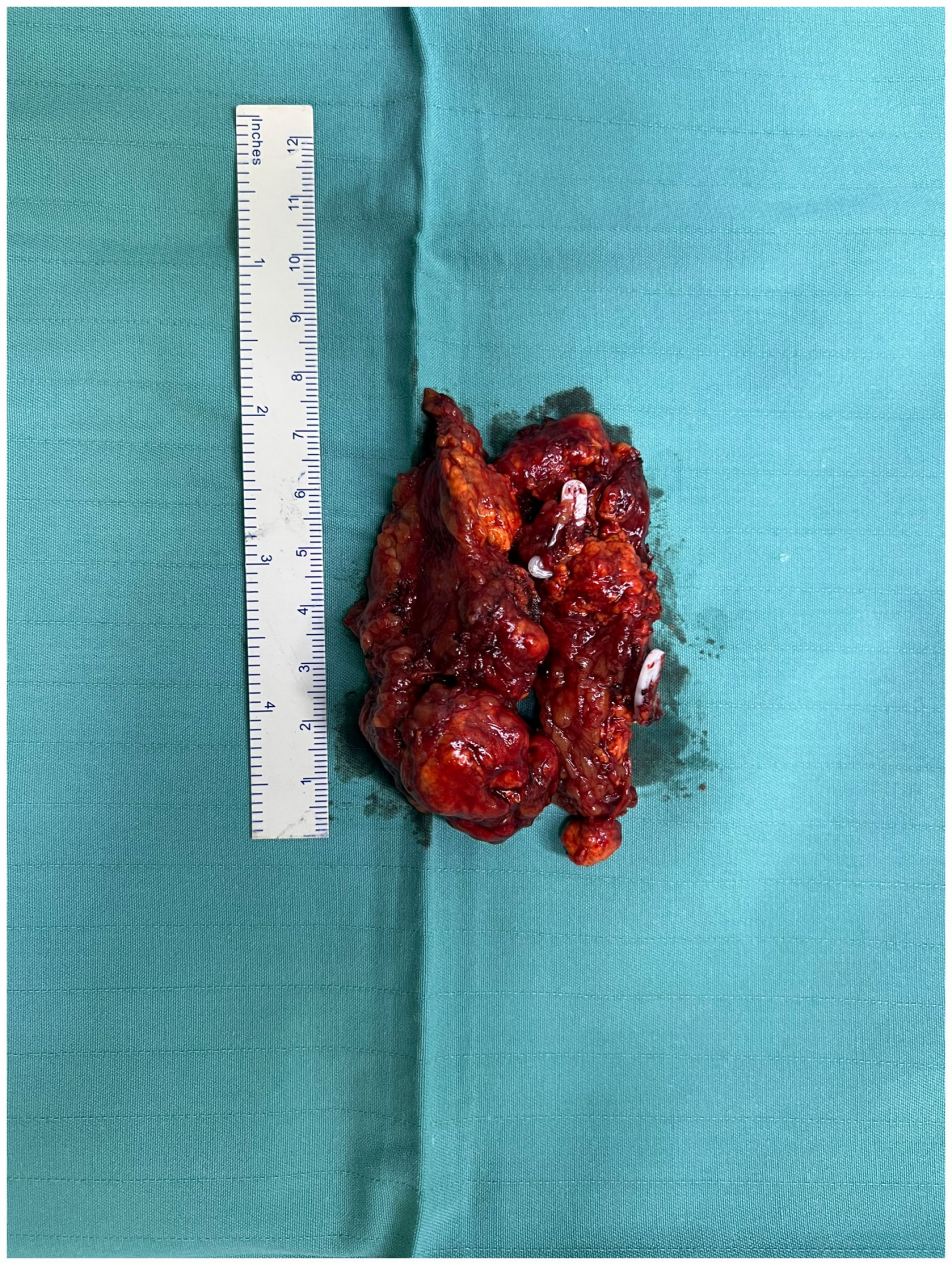
Gross pathological specimen of the resected left adrenal gland.

Histopathology (Figure 2): thickening of zona glomerulosa, fasciculata, and reticularis, with compact cell arrangement in the fasciculata, consistent with nodular adrenal hyperplasia. Immunohistochemistry: positive for Inhibin and Vimentin; Ki-67 ~ 5%; negative for CK, Melan-A, Synaptophysin, Chromogranin A, NSE, and CD10; p53 wild-type.

**Figure 3:**
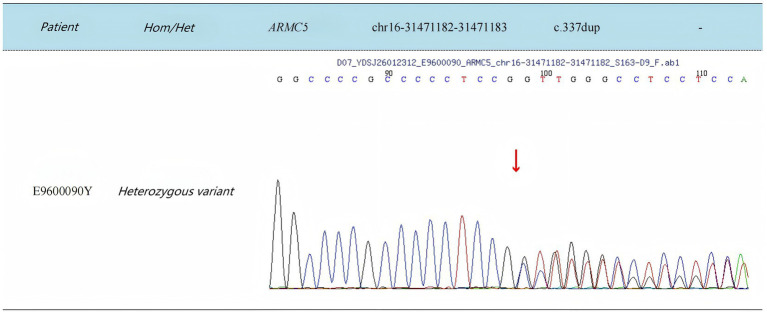
Sanger sequencing chromatogram confirming the ARMC5 gene variant.

Peripheral blood DNA analysis by Sanger sequencing identified a heterozygous germline frameshift insertion in ARMC5 (c.337dup) with a variant allele frequency of approximately 50% (Figure 3). Whole-exome sequencing of the resected adrenal tissue revealed an additional somatic frameshift deletion in ARMC5 (c.277_316del, p. P93fs) with a variant allele frequency of 43.1%, absent in the blood, demonstrating biallelic inactivation. No pathogenic mutations were found in KCNJ5, CACNA1D, ATP1A1, ATP2B3, or CTNNB1.

Family history: parents deceased, history unknown. The patient’s son tested negative for ARMC5 mutation, with normal blood pressure and glucose. Her elder sister was admitted on March 30, 2026, with hypertension, hypokalemia, and diabetes; CT showed bilateral adrenal macronodules similar to the proband. The sister underwent left adrenalectomy on April 2, 2026, with postoperative normalization of potassium. Regular follow-up is planned for all three.

## 3 Discussion

The combination of bilateral adrenal macronodules and isolated PA without hypercortisolism prompted postoperative ARMC5 genetic testing. This case of ARMC5-mutated bilateral macronodular adrenocortical disease (BMAD) presenting with isolated primary hyperaldosteronism (PA) and normocortisolemia differs from the known clinical spectrum of this genetic disorder. The work by Assié et al. established that ARMC5 germline mutations are a primary genetic driver of BMAD, clinically characterized by ACTH-independent hypercortisolism ranging from overt Cushing’s syndrome (CS) to mild autonomous cortisol secretion (MACS) (9). This association has been confirmed in subsequent large cohort studies, which report that most patients with ARMC5-related BMAD present with some degree of glucocorticoid excess, with overt CS or MACS as common findings (10, 11). The present case, however, exhibited a biochemical profile dominated by renin-independent aldosterone hypersecretion with severe hypertension and hypokalemia, while baseline cortisol and ACTH levels remained within normal limits, and the response to a high-dose dexamethasone suppression test was negative. This contrasts with the typical presentation where elevated cortisol, suppressed ACTH, and a positive dexamethasone suppression test are central to the diagnosis (12).

The observed phenotype is not consistent with the presumed exclusive link between ARMC5 dysfunction and glucocorticoid pathway dysregulation. Whole-exome sequencing of the resected adrenal tissue confirmed a somatic ARMC5 frameshift deletion (c.277_316del, p. P93fs) with a variant allele frequency of 43.1%, which was absent in the patient’s blood, demonstrating biallelic ARMC5 inactivation in the adrenal nodules. While ARMC5 alterations cause significant somatic molecular heterogeneity within adrenal nodules, meaning that different cells within the same nodule or between different nodules may harbor different secondary mutations, leading to variable steroidogenic enzyme expression and functional phenotypes (13), the predominant clinical outcome has been linked to cortisol. Recent histopathological studies correlating ARMC5 status with adrenal morphology have identified specific architectural patterns, such as pseudo-glandular and trabecular arrangements, associated with pathogenic variants (14). Furthermore, steroidogenic enzyme profiling has revealed distinct immunohistochemical clusters in BMAD. While Cluster 1, associated with ARMC5 mutations, shows reduced expression of enzymes like CYP11B1, Cluster 3, which comprises wild-type samples, is characterized by slightly increased CYP11B2 (aldosterone synthase) staining (15). This immunohistochemical evidence suggests an inverse correlation between inactivating ARMC5 mutations and CYP11B2 expression. We hypothesize that in this case, the somatic second hit (confirmed as c.277_316del) or the pattern of clonal expansion within the adrenal cortex preferentially affected the zona glomerulosa or its regulatory pathways, leading to a zona glomerulosa-dominant phenotype (16). This hypothesis is supported by the pathological finding of thickened adrenal zones, including the glomerulosa, and the postoperative normalization of aldosterone following unilateral adrenalectomy. The patient’s blood pressure had been well-controlled until 1 month before admission, when hypokalemia and worsening hypertension developed, suggesting that acquisition of the somatic ARMC5 second hit may have accelerated aldosterone production and clinical manifestation. The observed phenotype and the absence of other APA driver mutations suggest that biallelic ARMC5 inactivation may have directly or indirectly upregulated CYP11B2 in the zona glomerulosa. Our findings of renin-independent aldosterone excess in a patient with biallelic ARMC5 inactivation align with the proposed role of ARMC5 in predisposing to low-renin hypertension (17).

A mechanistic question is how the germline ARMC5 mutation relates to the somatic driver mutations of autonomous aldosterone synthesis (e.g., KCNJ5, CACNA1D, ATP1A1, ATP2B3). In aldosterone-producing adenomas (APAs), these somatic mutations directly activate CYP11B2 and aldosterone production, with reported frequencies of 37–43% for KCNJ5 and 10–21% for CACNA1D in large cohorts (18). In contrast, ARMC5 is a tumor suppressor gene whose biallelic inactivation drives bilateral macronodular adrenocortical disease (BMAD), classically associated with hypercortisolism (19, 20). The present case, however, presents isolated PA with normocortisolemia, and whole-exome sequencing of the adrenal tissue did not detect any pathogenic mutations in KCNJ5, CACNA1D, ATP1A1, ATP2B3, or CTNNB1.

Several hypothetical frameworks may explain how the germline ARMC5 mutation relates to aldosterone excess. First, the ARMC5 mutation may provide a permissive background of bilateral macronodular hyperplasia (20). Within this background, a somatic mutation in a classic PA driver gene (e.g., KCNJ5) could arise in a single nodule. The germline ARMC5 defect may hasten the proliferation of cells that acquire such a somatic aldosterone-driving mutation, leading to clonal expansion and focal aldosterone excess, a “two-hit plus” model supported by the known intraglandular heterogeneity of BMAD (13). Second, ARMC5 dysfunction alone might disturb adrenocortical zonation or cell lineage commitment, and recent cases have reported ARMC5 mutations with aldosterone co-secretion, raising the possibility that ARMC5 dysfunction alone can dysregulate aldosterone synthase (21, 22). These observations highlight the need for integrated molecular profiling in future cases.

Our case shows that BMAD is in the differential diagnosis for patients with bilateral adrenal nodules, even when the hormonal profile suggests isolated mineralocorticoid excess (23). Relying solely on the classic hypercortisolism phenotype may miss genetic diagnoses, as screening for primary aldosteronism (PA) is already underutilized in hypertensive populations (24). Our observation is consistent with a recent case report suggesting that ARMC5 mutation-associated BMAD may contribute to the development of PA, further supporting that this association represents a distinct clinical entity (25). From a pathophysiological perspective, this case differs from the presumed exclusive link between ARMC5 mutations and glucocorticoid hypersecretion. Emerging molecular evidence suggests that ARMC5 functions as a tumor suppressor involved in critical cellular processes, including the regulation of redox homeostasis through NRF1 turnover and the control of RNA polymerase II subunit degradation (26, 27). Specific somatic alterations or the pattern of clonal expansion in this patient may have disrupted adrenal cortical zonation or signaling pathways, such as Wnt/*β*-catenin, that favor aldosterone-producing cell lineage proliferation over glucocorticoid-producing cells (28).

The management of this patient with unilateral adrenalectomy for a bilateral disease process raises clinical questions. While the surgery was curative for hyperaldosteronism in the short term, the long-term risk of contralateral disease progression or emergence of hypercortisolism is uncertain. The role of unilateral versus bilateral adrenalectomy in BMAD is an area of active discussion, with recent studies indicating unilateral resection can be effective in selected cases (29). This decision is often guided by the asymmetry of adrenal involvement, yet predicting postoperative hormonal outcomes remains challenging. The potential for disease evolution, as seen in other forms of bilateral adrenal hyperplasia where a unilateral aldosterone-producing adenoma can emerge over time, warrants long-term follow-up (30). This case expands the clinical spectrum of ARMC5-related disease and highlights the need for comprehensive genetic testing in patients with bilateral adrenal enlargement, irrespective of the presenting steroid excess pattern.

The primary limitation of this report is that it is a single case, and the lack of long-term follow-up data limits definitive conclusions about the risk of contralateral progression or future hypercortisolism. We did not perform intranodular microdissection to assess clonal heterogeneity. Future studies are needed to corroborate this association and to elucidate the molecular mechanisms by which ARMC5 dysfunction may preferentially dysregulate aldosterone synthesis.

## Data Availability

The datasets presented in this study can be found in online repositories. The names of the repository/repositories and accession number(s) can be found at: http://www.ncbi.nlm.nih.gov/bioproject/1420368, PRJNA1420368.
